# Impaired Renal Function Due to Reductive Stress Is Associated with Excessive Consumption of *Hibiscus sabdariffa Linnaeus*

**DOI:** 10.3390/antiox14080984

**Published:** 2025-08-11

**Authors:** Linaloe Manzano-Pech, María Elena Soto, Vicente Castrejón-Tellez, Verónica Guarner-Lans, Alan Axel Pérez-Flores, Sara Caballero-Chacón, Raúl Martínez-Memije, María Esther Rubio-Ruiz, Félix Leao Rodríguez-Fierros, Juan Carlos Torres-Narváez, Luz Ibarra-Lara, Israel Pérez-Torres

**Affiliations:** 1 Department of Physiology and Pharmacology UNAM, Facultad de Medicina y Veterinaria y Zootecnia, Av. Universidad 3000, Coyoacán 04510, Mexico; loe_mana@hotmail.com (L.M.-P.); paxel89@live.com.mx (A.A.P.-F.); saracachas@hotmail.com (S.C.-C.); 2 Research Direction, Instituto Nacional de Cardiología Ignacio Chávez, Juan Badiano 1, Sección XVI, Tlalpan, Mexico City 14080, Mexico; mesoto50@hotmail.com; 3 Department of Physiology, Instituto Nacional de Cardiología Ignacio Chávez, Juan Badiano 1, Sección XVI, Tlalpan, Mexico City 14080, Mexico; vicente.castrejon@cardiologia.org.mx (V.C.-T.); veronica.guarner@cardiologia.org.mx (V.G.-L.); esther_rubio_ruiz@yahoo.com (M.E.R.-R.); 4 Department de Electromechanical Instrumentation, Instituto Nacional de Cardiología Ignacio Chávez, Mexico City 14080, Mexico; raulmmemije@yahoo.com; 5 Laboratorio de Patología Veterinaria, Facultad de Ciencias Naturales, Universidad Autónoma de Querétaro, Santiago de Querétaro 76230, Mexico; felix.rodriguez@uaq.mx; 6 Department de Pharmacology, Instituto Nacional de Cardiología Ignacio Chávez, Juan Badiano 1, Sección XVI, Tlalpan, Mexico City 14080, Mexico; juancarlostn63@hotmail.com (J.C.T.-N.); luz.ibarra@cardiologia.org.mx (L.I.-L.); 7 Department of Cardiovascular Biomedicine, Instituto Nacional de Cardiología Ignacio Chávez, Mexico City 14080, Mexico

**Keywords:** HSL, reactive oxygen species, antioxidant enzymes, reductive stress, kidney, redox couples

## Abstract

Reductive stress (RS) results from the overactivity of the enzymatic and non-enzymatic antioxidant systems and from excess antioxidant agents that neutralize reactive oxygen species. *Hibiscus sabdariffa Linnaeus* (HSL) is a natural source of antioxidant molecules that can overload the antioxidant system. Twenty-one Wistar rats were divided into three groups: group 1 (G) G1: rats that consumed a 6% HSL infusion for one month (HSL + 6%), G2: rats that consumed a 6% HSL infusion for one month and were then given natural water for another month (HSL ± 6%), and G3: rats with natural drinking water. Renal vascular resistance (RVR) was evaluated through their responses to norepinephrine (Ne), acetylcholine (Ach), super oxide (O_2_^−^), hydrogen peroxide (H_2_O_2_)_,_ and peroxynitrite (ONOO^−^). The activity of antioxidant enzymes and oxidative stress markers was evaluated. RVR was increased by Ne and H_2_O_2_ (*p* = 0.03), but it was decreased by Ach, O_2_^−^, and ONOO^−^ (*p* = 0.01). The reduced glutathione / oxidized glutathione (GSH/GSSG) ratio and nitrates/nitrites ratio, the total antioxidant capacity, the activities of superoxide dismutase, catalase, peroxidases, glutathione peroxidase, glutathione reductase, glucose-6-phosphate, and the expression of phosphorylated NrF2 were increased (*p* ≤ 0.04). However, the thiol groups, adenochrome, and glutathione-S-transferase were decreased (*p* = 0.01) in G1 vs. G2 and G3. The excessive consumption of antioxidants provided by a 6% HSL infusion results in RS contributing to a decrease in ROS.

## 1. Introduction

Redox balance is essential for maintaining cellular homeostasis. A moderate production of reactive oxygen species (ROS) leads to beneficial effects since these molecules act as second messengers [1]. However, overproduction results in oxidative stress (OS) [2].

The superoxide anion (O_2_^−^) is a ROS that is formed by the univalent reduction in O_2_ by various enzymes within the cell [3]. Once formed, it rapidly dismutates to H_2_O_2_ through enzymatic and non-enzymatic mechanisms. H_2_O_2_ is capable of easily crossing cell membranes in high levels, and it is cytotoxic in cultured cells [4]. The rate of the reaction that produces it is only limited by the diffusion coefficient of the molecule in the presence of SOD isoforms. Intracellular H_2_O_2_ levels are tightly regulated by different enzymes responsible for oxidizing it into water and molecular O_2_, such as catalase (CAT), glutathione peroxidase (GPx), and peroxidases [5,6]. The O_2_^−^ that does not dismutate is less reactive with proteins, DNA, and lipids, and it mainly reacts with nitric oxide (NO) to form peroxynitrite (ONOO^−^) and with transition metals. Therefore, it practically only reacts with proteins containing iron–sulfur centers [5]. However, H_2_O_2_ is more stable than O_2_^−^ and the hydroxyl radical (OH^−^) [5,6]. In addition, other ROS include peroxynitrites (ONOO^−^), alkoxyl radical (RO^•^), peroxy radical (ROO^•^), hypochlorous acid (HOCl^−^), nitric oxide (NO), and nitric monoxide (^•^ON), which are generated in peroxisomes, the plasma membrane, the endoplasmic reticulum, and the cytoplasm [7]. NO and ^•^ON act as messenger molecules that contribute to aortic vasodilation [8]

A moderate production of ROS is important in different metabolic pathways because these molecules act as second messengers, modifying post-translational protein conformation. They regulate the stability and activity of proteins, and they modulate cellular functions [9]. H_2_O_2_ modulates signal transduction by reversible oxidation of cysteines that have redox activity [10]. Moreover, H_2_O_2_ can activate at least 40 gene products in mammalian cells [11]. Furthermore, the non-cytotoxic level of ROS (pM concentrations) is ensured by the enzymatic and the non-enzymatic antioxidant systems, which participate in the homeostasis of cellular redox processes [12]. If the redox balance is modified by 30 millivolts, it represents a 10-fold change between the proportion of reducing species, such as the GSH/GSSG ratio, and pro-oxidants, such as O_2_^−^ [13].

However, decreased ROS, excessive antioxidant molecules, and/or overproduction of the enzymatic and non-enzymatic antioxidant systems lead to reductive stress (RS) with pathological consequences [2]. RS is a concept that is currently not well defined; however, it may result in detrimental effects in a similar way to that of OS [14]. RS is characterized by an excess of reducing equivalents in the form of redox couples, such as reduced nicotinamide adenine dinucleotide/oxidized nicotinamide adenine dinucleotide (NADPH^+^/NADP^+^), NAD^+^/NADH^+^, and GSH/GSSG, or the overexpression of the antioxidant enzymatic systems, which can deplete ROS [15]. Increases in these reducing equivalents and enzymatic and non-enzymatic antioxidant systems have been reported [15], but the mechanisms of action, the cellular responses involved, and the biological consequences of these changes remain unknown. In this regard, excess reducing equivalents decrease cell growth, induce alteration in the formation of disulfide bonds between proteins, reduce mitochondrial function, decrease cellular metabolism, and contribute to the development of some diseases that are closely associated with inflammatory conditions, such as protein aggregation cardiomyopathy, hypertrophic cardiomyopathy, muscular dystrophy, pulmonary hypertension, rheumatoid arthritis, cancer, and metabolic syndrome, among others [13,14,15].

Chronic RS can also induce OS by a positive feedback regulation [15]. NADPH^+^ is a cofactor responsible for donating protons in various reactions catalyzed by enzymes. NADH^+^ overproduction puts pressure on the mitochondrial complex I, which responds within its capacity to oxidize and converts NADH^+^ to NAD^+^. This event causes increased electron leakage and, consequently, decreases the oxygen available to produce O_2_^−^. However, elevated NADH^+^ levels favor an oxidizing environment but also achieve the transition to RS via the polyol pathway (PPP) [14]. This pathway is an alternative route of glucose metabolism, where the conversion of glucose to glucose-6-phosphate by glucose-6-dehydrogenase (G6PD) depends exclusively on the available concentration of unphosphorylated glucose [16]. In addition, nuclear factor erythroid-derived 2 (Nrf2) signals the transcription of antioxidant enzymes [17], such as the superoxide dismutase isoforms (SODs), glutathione-s-transferase (GST), GPx, and glutathione reductase (GR) [18,19]. Despite the above, RS is a poorly explored field of study.

Regarding the enzymatic antioxidant system, SOD isoforms are metalloenzymes utilizing metal cofactors that catalyze the reduction of the free radical O_2_^−^ to O_2_ and H_2_O_2_. SOD isoforms are classified according to their metal cofactors and location, being divided into copper–zinc (Cu-Zn) SODs located in the cytoplasm, manganese (Mn) SODs located in the mitochondria, and extracellular (-ex) SODs found in plasma [20].

The enzymatic antioxidant system also comprises enzymes that use selenium (Se). One of them is the GPx, which catalyzes the reduction of H_2_O_2_ and/or organic hydroperoxides to water or alcohol, respectively, using GSH as the reducing agent [21]. Another Se enzyme is thioredoxin reductase (TrxR), which is responsible for reducing thiol groups between protein cysteines and for providing electrons to thiol-dependent peroxidases, using NADPH^+^ as a cofactor [22,23,24,25]. Another Se enzyme is GST, which is involved in the detoxification of xenobiotics and in protection against damage caused by peroxides. It catalyzes the ionization of GSH to the nucleophilic thiolate anion (GS-), which is capable of spontaneously reacting with closely related nucleophilic components, followed by substrate conjugation. This conjugation increases the solubility of the product, facilitating its excretion from the cell [26]. GR is a flavoprotein that catalyzes the reduction of GSSG to GSH. It is a critical molecule in the defense against OS by maintaining a reducing environment in the cell. A decrease in the activity of this enzyme leads to a decrease in GSH and the accumulation of GSSG [27]. Other enzymes that are not Se-dependent but are utilized to reduce H_2_O_2_ are the peroxidases that decompose various hydroperoxides (ROOH, mainly H_2_O_2_) to oxidize organic and inorganic substrates [28]. CAT, another member of the enzymatic antioxidant system, is involved in the destruction of H_2_O_2_ [29].

In addition to the enzymatic antioxidant system, there is the non-enzymatic antioxidant system, which is constituted by a series of compounds that delay the production and the action of ROS, and include GSH, flavonoids, resveratrol, and polyphenols, among others. GSH is a tripeptide composed of three amino acids: cysteine, glycine, and glutamic acid [30]. It is a soluble antioxidant found in cellular compartments, and it protects cells from oxidative damage to lipids, proteins, and nucleic acids. GSH acts by trapping OH^−^, O_2_^−^, and ONOO^−^ and by reactivating enzymes that are inhibited at high oxygen concentrations, such as TrxR and GST [31]. In the presence of ROS, GSH is oxidized to GSSG and subsequently reduced by GR to GSH; therefore, the GSH/GSSG ratio is a marker of cellular toxicity. However, a normal level of this index is essential for some cellular functions, such as cell proliferation, differentiation, and apoptosis [32]. However, if increased, it decreases ROS and favors RS, affecting cellular pathways [15].

On the other hand, *Hibiscus sabdariffa Linnaeus* (HSL), also known as Jamaica flower, is native to tropical Africa [33], and it is used in gastronomy in various foods, such as beverages, sweets, and jams. Nevertheless, there is no well-founded consensus on the percentage of HSL used in the formulations of many beverages, which may range from 0.5 to 1%. In traditional medicine, HSL is used to treat pyrexia, hypertension, liver damage, and kidney diseases, and it reduces cholesterol and blood pressure. There is a background of OS in these pathologies, and the polyphenols, anthocyanins, protocatechuic acid, epigallocatechins, resveratrol, and flavonoids provided by HSL reduce these conditions [34,35]. Therefore, evidence suggests that HSL infusion at low concentrations can be used as an alternative medicinal procedure to improve several pathologies, due to the high antioxidants that it provides; however, chronic and excessive consumption in clinically healthy humans and animals may have adverse effects, which are still matters of investigation. In a different context, the function of the kidney is to remove toxic substances produced by cellular metabolism through glomerular filtration. It also regulates blood pressure, hemodynamics, and the acid–base balance [36]. The fundamental unit of the kidney is the nephron, made up of the renal corpuscles, which are integrated by the glomerulus, Bowman’s capsule, the afferent and efferent arterioles [37], the proximal and distal tubules, the loop of Henle, and the distal tubule. These arterioles in the glomerulus receive sympathetic (adrenergic) and parasympathetic (cholinergic) innervation. Sympathetic activity releases norepinephrine (Ne), which contributes to vasoconstriction, regulates blood flow and the glomerular filtration rate, and the Na^+^ reabsorption of water through adrenergic receptors [38]. In contrast, the parasympathetic system activates eNOS, which generates NO, which acts on soluble guanylate cyclase, decreasing both afferent and efferent arterial resistance, being one of the physiological mechanisms resulting in renal vasodilation through the secretion of acetylcholine (Ach) [39,40]. Therefore, the evaluation of whether a decrease in ROS could lead to RS is justified since this condition is associated with increased redox equivalents and/or overexpression of the enzymatic and non-enzymatic antioxidant system. The chronic consumption of HSL, which is a natural source of antioxidants that promotes the overexpression of the enzymatic and non-enzymatic antioxidant system and decreases ROS, could act as a tool to evaluate the presence of RS in the kidney. Moreover, there are few studies on whether the chronic and excessive consumption of high concentrations of HSL induces toxic effects.

Therefore, the aim of this study was to evaluate the effect of chronic consumption of a 6% HSL infusion on the potential alteration of the enzymatic and non-enzymatic antioxidant system associated with probable renal damage and the possible generation of RS in male Wistar rats.

## 2. Materials and Methods

### 2.1. Rat Groups

This study was designed and carried out in compliance with the Laboratory Animal Care Committee of the National Institute of Cardiology Ignacio Chávez (INC/CICUAL/009/2023) approved for experiments in animals. The experiments were conducted in compliance with the Guide for the Care and Use of Laboratory Animals of the National Institutes of Health (NIH). Twenty-one male Wistar rats were used to form three groups with seven animals each, as follows: Group 1 rats received ad libitum a 6% HSL infusion for one month (HSL 6%). Group 2 rats received ad libitum a 6% HSL infusion for one month, after which they were given tap water for another month (HSL ± 6%). Group 3 rats received plain tap water ad libitum for one month (C). The animals were housed for 4 weeks under the following conditions: a 12 h light/12 h dark cycle, ambient temperature, and relative humidity ranging from 18–26 °C to 40–70%, respectively. The commercial food the rodents consumed was solid rodent kibble supplemented with 23% crude protein, 4.5% crude fat, 6% crude fiber, 8% ash, and 2.5% minerals (Labdiet 5008; PMI Nutrition International, Richmond, IN, USA) ad libitum.

### 2.2. Preparation of the HSL Infusion

A total of 60 g of HSL calyces were added to one liter of boiling water. It was kept boiling for 10 min, allowed to cool, and filtered. This solution was provided ad libitum to the rats.

### 2.3. Calculation for the Sample Size

The mean systolic blood pressure (SBP) in healthy rats has been reported to be between 116 mmHg and 112.8 mmHg, with a variance of 19, in rats from the National Institute of Cardiology Ignacio Chavez. Based on this, the sample size per group was estimated by μ (SBP of Wistar rats), with 95% confidence and a maximum error (ME) of 3.2 mmHg, according to the following formula:

ME:|μ−x¯|=|116−112.8|=3.2 mmHgConfidence:|μ−x¯|=|116−112.8|=3.2SS:n=(σ¯x)2σ2x/(EM2)=(22)×(22)/(3.2)2=(4×19)/(10.24)=76/10.24=7.42

where ME = maximum error, ${\bar{\sigma }}_{x}=\#$ of standard deviations of the mean estimator, σ^2^_x_ = variance in the SBP of the rats in our institute, and SS = sample size.

### 2.4. Blood Sample Obtainment and Measurement of Renal Function

Blood was obtained directly from the aorta prior to performing the isolated and perfused kidney technique. It was centrifuged at 3000 rpm for 20 min at 4 °C. Serum was obtained. The SCr and UCr levels were determined. Using the collected 24 h urine volume, the UCr clearance was calculated using the following formula: CCr = (SCr/UCr (Urine Volume)/1440 min. For a detailed description of the methodology used for both the isolated and perfused kidney and the enzymatic and non-enzymatic antioxidant system, please refer to the Supplementary Material.

### 2.5. Nuclear Factor Erythroid 2 (NrF2)

A total of 50 μg of protein from the kidney homogenate was separated on an 8% SDS-PAGE gel and transferred to polyvinylidene difluoride membranes. The blot was blocked for 1 h at room temperature using Tris-buffered saline (TBS)-0.01% Tween plus 5% skim milk. The membranes were incubated overnight at 4 °C with the primary anti-phospho-NrF2-S40 monoclonal antibody produced in rabbit SAB5701902-100UL. The blot was then incubated with the β-actin antibody (sc-81178) as a loading control. The membranes were analyzed by densitometry, using a GS-800 densitometer with Quantity One 1-D analysis software version 4.6.8 (Bio-Rad Laboratories, Inc., Hercules, CA, USA), and were reported as arbitrary units (AUs).

### 2.6. Statistical Analysis

Statistical analysis and graphs were performed using Sigma Plot software (SigmaPlot^®^ version 15.0, Jamdel Corporation, Boca Raton, FL, USA). Data are presented as mean ± standard error. Statistical significance was determined using one-way ANOVA and Tukey’s post hoc test. A *p* ≤ 0.05 was considered significant.

## 3. Results

### 3.1. Guaranteed Analysis

The 6% HSL infusion contained cyanidin-3-glucoside (495.40 ± 43.47 mg/L), quercetin (148.48 ± 15.13 mg/L), and polyphenols (27.65 ± 0.76 mol/L).

### 3.2. General Characteristics and Markers of Kidney Function

Table 1 shows the general characteristics and markers of renal function in the rats in the three experimental groups. The rats receiving the 6% HSL infusion showed decreased water consumption, 24 h urine volume, and CCr clearance, but increased albuminuria and SBP compared to the C and HSL ± 6% groups (*p* = 0.001). However, in the HSL ± 6% group, the significant difference in CCr clearance and SBP was lower than in the C group (*p* = 0.01).

### 3.3. Markers in the Non-Enzymatic Antioxidant System

Table 2 shows the levels of some markers in the non-enzymatic antioxidant system in the experimental groups. The rats receiving the 6% HSL infusion showed significant increases in the GSH/GSSG and NO_3_^−^/NO_2_^−^ ratios, total antioxidant capacity, and thiol groups compared to the C and HSL ± 6% groups (*p* = 0.01). However, the total antioxidant capacity and thiol groups showed significant differences between the C and HSL ± 6% groups (*p* = 0.02).

### 3.4. Changes in the Δ-PP in the Kidney

**Figure 1:**
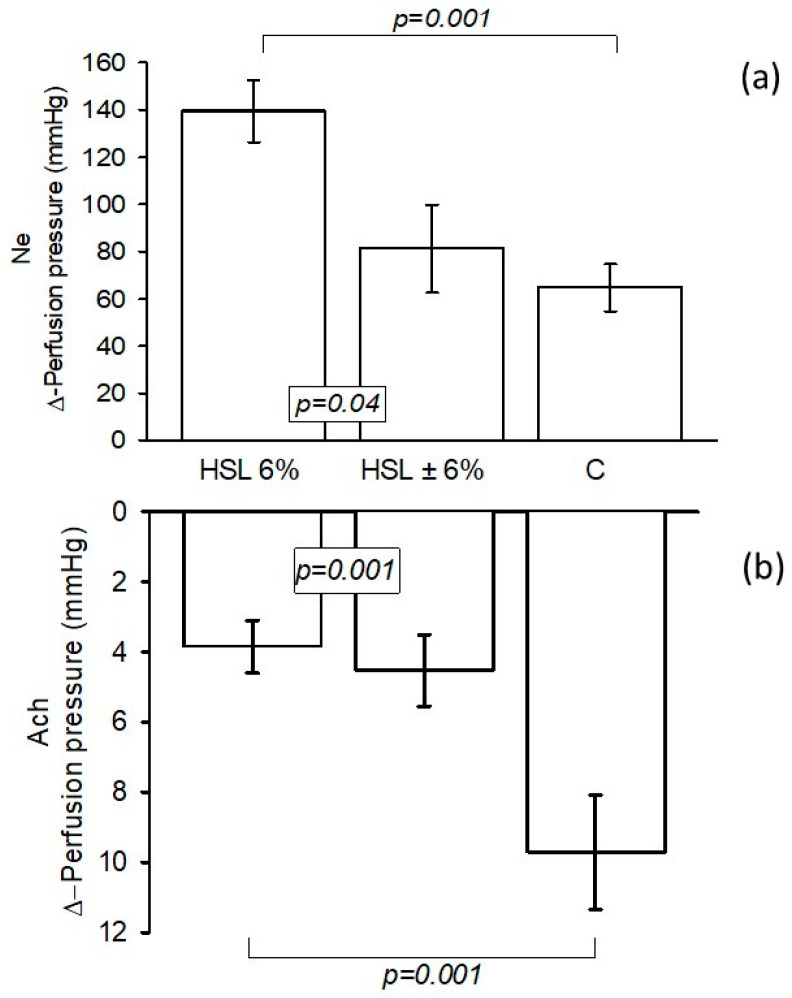
Values of the Δ-PP in the kidney exerted by Ne on renal vascular resistance (**a**) and the Δ-PP in the kidney exerted by Ach on renal vascular resistance (**b**). (n = 7), the results expressed as the mean ± standard error.

Figure 1a shows the changes in the Δ-PP in the kidney, which increased significantly (*p* = 0.001) when 20 μM Ne was perfused into the kidney of the rats that consumed the 6% HSL infusion compared to the rats in group C. Eliminating the HSL treatment for one month in the HSL ± 6% rat group showed a significant reduction (*p* = 0.04). Figure 1b presents the changes in the Δ-PP in the kidney when infusing 20 μM of Ach. The results showed that the group of rats that consumed the 6% HSL infusion had increased vascular resistance in Δ-PP compared to the control group and the HSL group ± 6% (*p* = 0.001), since there was greater vasodilation in these last groups.

**Figure 2:**
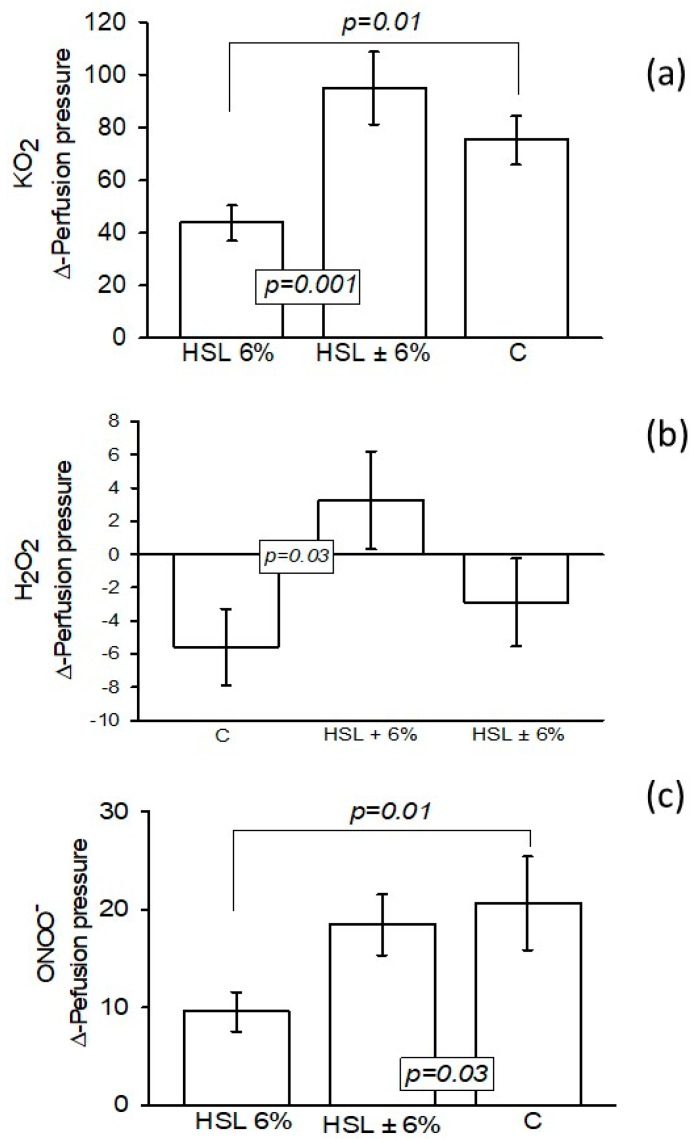
The values of the Δ-PP in the kidney when exposed to O_2_^−^ (**a**), H_2_O_2_ (**b**), and ONOO^−^ (**c**) on renal vascular resistance in the experimental groups (n = 7), with the results expressed as the mean ± standard error.

When 2 μM KO_2_ was infused into the kidneys from the rats consuming the 6% HSL infusion, the Δ-PP was significantly decreased compared to the control and HSL ± 6% groups (*p* = 0.01 and *p* = 0.001, respectively, as shown in Figure 2a. When 1.7 μM H_2_O_2_ was infused into the kidneys from rats consuming the 6% HSL infusion, the Δ-PP was significantly increased compared to group C (*p* = 0.03) but remained unchanged with respect to group HSL ± 6% (Figure 2b). When ONOO^−^ was infused into the kidneys from the rats consuming the 6% HSL infusion, the Δ-PP was significantly decreased compared to groups C and HSL ± 6% (*p* = 0.01 and *p* = 0.03, respectively, as shown in Figure 2c.

### 3.5. Anatomical Changes in the Kidney

**Figure 3:**
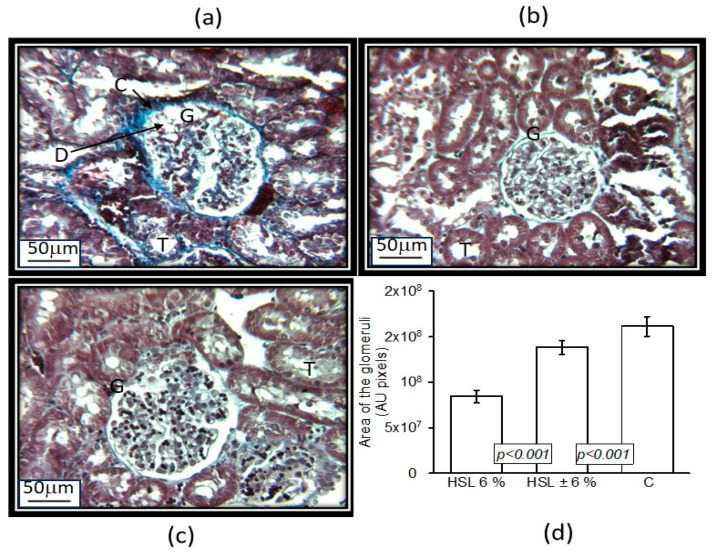
Panels (**a**–**c**) show the representative histological sections of the glomerulus in each of the 3 experimental groups. Panel (**d**) shows the area of the glomeruli analyzed by densitometry in the experimental groups. Masson’s trichrome staining makes collagen fibers evident in blue. Masson’s trichrome technique (25×). Abbreviations: C = collagen, D = debris, G = glomerulus, and T = tubules. Panels (**a**) = HSL 6%; (**b**) = HSL ± 6%; (**c**) = control group; and (**d**) densitometry area.

**Figure 4:**
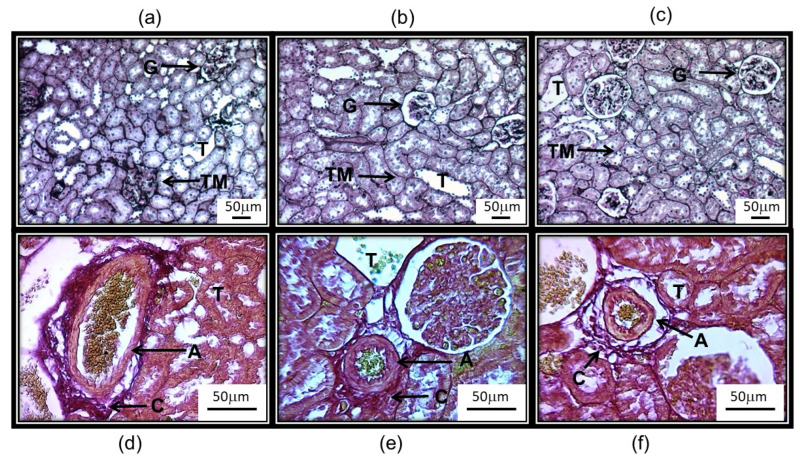
Panels (**a**–**c**) show the representative histological sections of the tubules in each of the 3 experimental groups, where it is observed that there is a thickening of the tubular membrane in the tubular interstitium in the rats that consumed the 6% HSL infusion. Jones’s methenamine technique (12.5×). The same figure, but on panels (**d**–**f**), shows the arterial vessels in each of the 3 experimental groups, where it is observed that there is perivascular fibrosis with thickening of the adventitia of the vessels in the rats that consumed the 6% HSL infusion in comparison to the C and HSL ± 6% rats. Sirius red technique (32×): Sirius red staining makes the collagen fibers evident in red around the adventitia of the vessel. Abbreviations: G = glomerulus, T = tubules, TM = tubules membrane, A = adventitia, and C = collagen. Panels (**a**,**d**) = HSL 6%; (**b**,**e**) = HSL ± 6%; and (**c**,**f**) = control group.

Figure 3 panels a, (6% HSL infusion), b, (HSL ± 6%), and c, (C group) show the representative histological sections of a glomerulus from each of the experimental groups of rats. The rats that consumed the 6% HSL infusion presented retraction of the glomerular tuft, fibrosis, cellular debris, and an increase in urinary space compared to groups C and HSL ± 6%. Panel d in Figure 3 shows the photometric density analysis of the area of the glomeruli in the experimental groups. There was a smaller area in the group of rats that consumed the 6% HSL infusion compared to group C (*p* < 0.001). In the group of HSL ± 6% rats, there was no significant difference compared to the control group. Figure 4, panels (a, 6% HSL infusion), (b, HSL ± 6% rats), and (c, C group) show the representative histological sections of tubules where there was a thickening of the tubular membrane in the tubular interstitium in the rats that consumed the 6% HSL infusion in comparison to the C and HSL ± 6% rats. The same figure in panels (d–f) shows the arterial vessels in each of the three experimental groups, where it is observed that there is perivascular fibrosis with thickening of the adventitia in the rats that consumed the 6% HSL infusion in comparison to the C and HSL ± 6% rats.

### 3.6. The Enzyme Activity of the Enzymatic Antioxidant System in Native Polyacrylamide Gels

**Figure 5:**
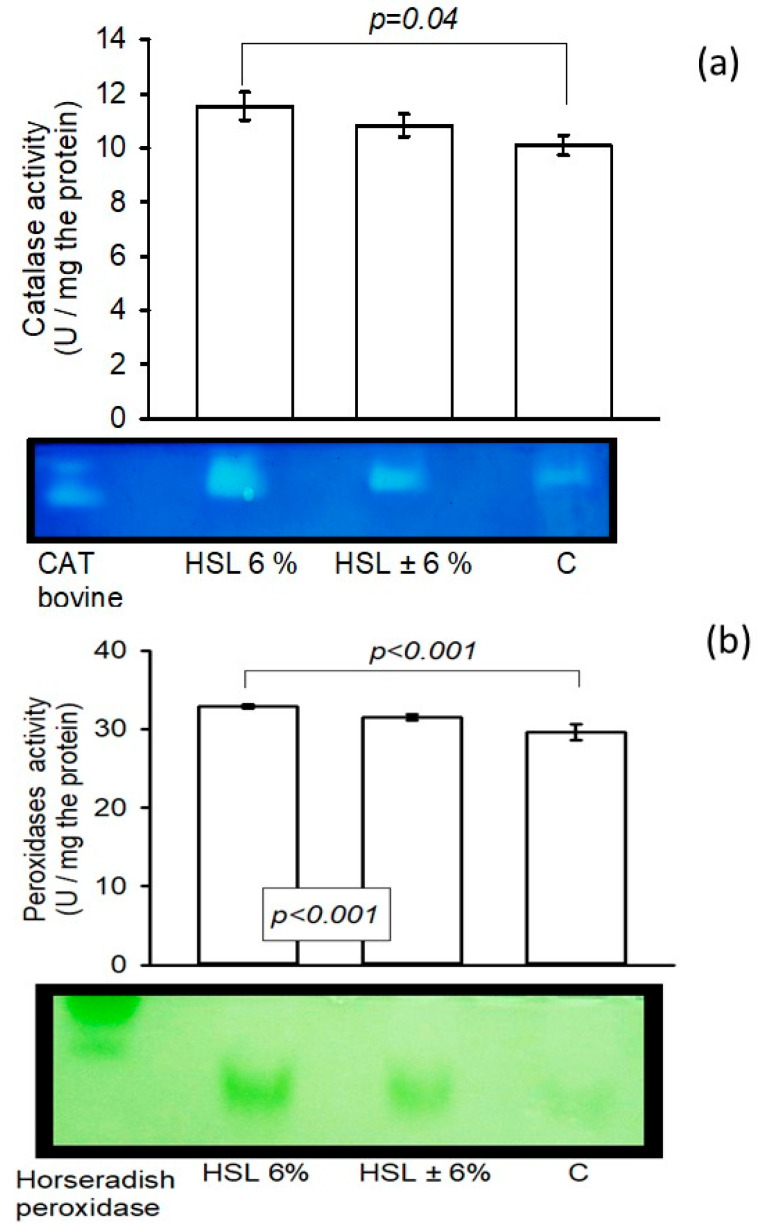
Enzymatic activities of CAT (**a**) and peroxidases (**b**) in an 8% native polyacrylamide gel. The images below the histograms represent the activity levels. The values are expressed as the mean ± standard error (n = 7).

Figure 5a shows the enzymatic activity of CAT in kidney homogenates. There is a significant increase in the group of rats that consumed the 6% HSL infusion (*p* = 0.04) compared to group C, but without a significant difference compared to the ±6% HSL group. The same figure presents in panel (b) the enzymatic activity of peroxidases in the kidney homogenate in the experimental groups, where there was a significant difference between the group that consumed the 6% HSL infusion compared to the HSL ± 6% groups and group C (*p* < 0.001).

**Figure 6:**
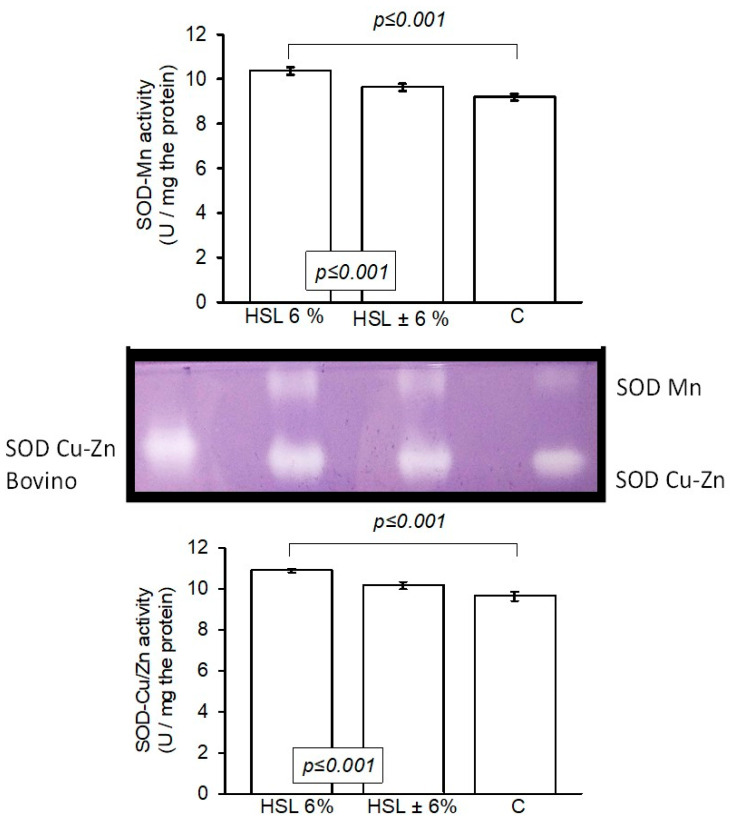
Enzymatic activity of SOD isoforms in an 8% native polyacrylamide gel. The image in the center of the histogram shows the activity of both isoforms. The values are expressed as the mean ± standard error (n = 7).

Figure 6 shows that the activity of SOD isoforms (Mn and Cu-Zn, respectively) was significantly increased in the rats that received the HSL 6% infusion (*p* ≤ 0.001) compared to the rats in the C and HSL ± 6% groups.

### 3.7. The Activity of GSH-Using Enzymes in the Enzymatic Antioxidant System by Spectrophotometry

**Figure 7:**
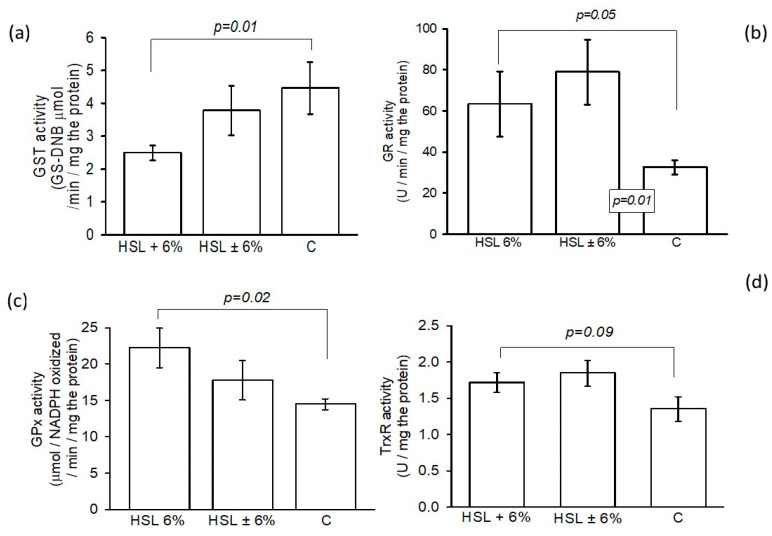
Activities of GST, GR, GPx, and TrxR (panels (**a**), (**b**), (**c**), and (**d**), respectively) of the antioxidant enzymes that employ GSH as a cofactor in the ROS detoxification process in the three experimental groups. The values are expressed as the mean ± standard error (n = 7).

Figure 7 shows the GST activity in panel (a), where a significant decrease was observed between the HSL + 6% group and group C (*p* = 0.01). However, no significant changes were observed in the HSL ± 6% group. Panel (b) shows differences in the activity of GR in the kidney homogenates between the experimental groups, where a significant increase was observed between the HSL 6% and HSL ± 6% groups (*p* = 0.05 and *p* = 0.01, respectively) compared to group C. The GPx activity showed a significant increase (*p* = 0.02) in the kidney homogenates in the group of rats that consumed the 6% infusion compared to the rats in group C, but no significant changes compared to the HSL ± 6% group C (c). Finally, panel (d) of the same figure shows the enzymatic activity of TrxR in the HSL + 6% group. There were no significant changes with respect to HSL ± 6%, but there was a tendency to decrease without significant changes (*p* = 0.09) in group C.

### 3.8. G6PD Activity and NrF2 Phosphorylated Expression

**Figure 8:**
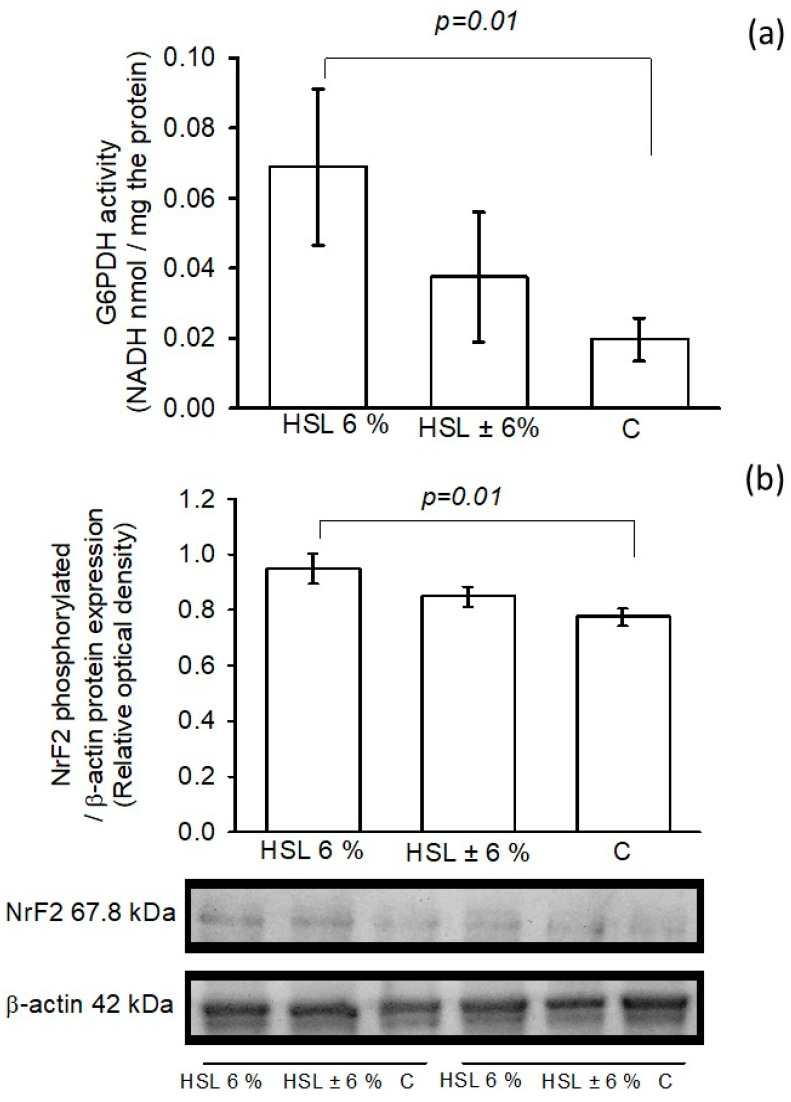
The G6PD activity (**a**) and the NrF2 phosphorylated/β-actin ratio expression (**b**) in the three experimental groups in the kidney homogenate. The values are expressed as the mean ± standard error (n = 7).

Figure 8a shows the G6PD activity in kidney homogenates in the three experimental groups. The group receiving the 6% HSL infusion showed a significantly increased G6PD activity (*p* = 0.01) compared to group C, but the activity remained unchanged when compared to the ± 6% HSL group. In the same figure, in panel (b), the phosphorylated expression of Nrf2 in the kidney homogenate in the three groups of rats is shown, where the group of rats that consumed the 6% HSL infusion showed a significant increase (*p* = 0.01) compared to group C, but without significant changes with respect to the HSL ± 6% group.

### 3.9. O_2_^−^ Anion Quantification

**Figure 9:**
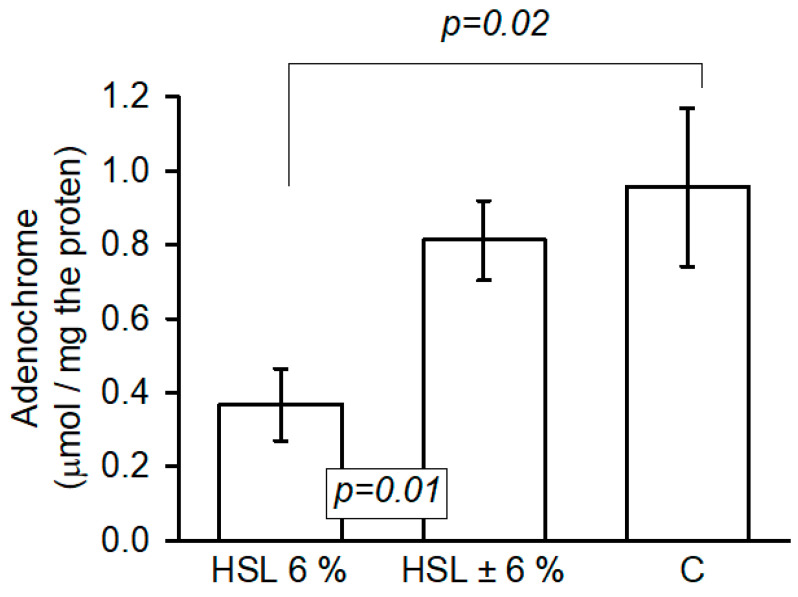
The indirect determination of O_2_^−^ anion production by the irreversible oxidation of epinephrine to adenochrome in kidney homogenates in the three experimental groups. The values are expressed as the mean ± standard error of the relative optical density (n = 7).

Figure 9 shows the oxidation of epinephrine to adenochrome by the action of the O_2_^−^ anion, which is an accepted method for measuring the generation of this radical. The results showed a significant decrease (*p* = 0.02 and *p* = 0.01, respectively) in the kidney homogenate of the rats in the group that consumed the 6% HSL infusion compared to groups C and HSL ± 6%.

## 4. Discussion

A reducing environment is associated with beneficial effects on cellular functions and biological processes, but chronic increases in the components of this environment could disrupt redox homeostasis and lead to detrimental effects on ROS signaling, participation, and production in different cellular pathways [41]. At normal concentrations, ROS play a key role in cellular defense, hormone synthesis and signaling, spermatogenesis, activation of G protein-coupled receptors, transcription factors, modulating vascular reactivity, regulating electrolyte balance in the renal tubules, and gene expression [42]. However, at high concentrations, they lead to OS.

On the other hand, HSL is a functional food because it provides various components with biological activity; for example, it provides antioxidant molecules, minerals, and essential amino acids. In the pathological conditions that have a highly oxidative background, such as metabolic syndrome and Marfan syndrome, 1.5–2% HSL infusions reduce OS, which is reflected in an improvement in redox homeostasis [34,43]. However, these beneficial characteristics could become harmful if HSL is consumed for a prolonged period of time and in excess. Its excessive consumption may promote an increase in the antioxidant enzymes and reducing equivalents, which could lead to RS and, consequently, to an imbalance in redox homeostasis that may lead to alterations in different organs, such as the kidneys. The balance between prooxidants and antioxidant molecules is essential for proper renal function [44].

Therefore, the objective of this study was to generate an RS state associated with excessive and chronic consumption of a 6% HSL infusion that could alter renal function. We also tried to demonstrate how a decrease in ROS due to the overexpression of the enzymatic and non-enzymatic antioxidant systems may contribute to the deterioration of renal physiology.

The results in this paper show an increase in Δ-PP in the presence of Ne, but a decrease with Ach. When the kidney was experimentally kept in a constant flow, a slight increase in the Δ-PP caused by both vasoconstrictors or vasodilators represents changes in the resistance in the afferent arteriole. According to the biogenic theory, which postulates that the renal blood flow is equal to the blood pressure divided by the renal vascular resistance (RVR), when the Δ-PP increases or decreases, the vessels modify the resistance. This mechanism is intrinsic and self-regulatory in normal renal conditions, but when there is an imbalance in pathological conditions, an increase or decrease in the resistance in the pre-glomerular afferent arteriole by the action of vasoconstriction or vasodilation modifies the glomerular filtration rate [45]. This contributes to the decrease in glomerular filtration rate, CCr clearance, and the presence of proteinuria in the rats that consumed the 6% HSL infusion vs. groups C and HSL ± 6%. Furthermore, these changes are associated with anatomical changes in the nephron, including fibrosis in renal arterioles and tubules, resulting in increased intraglomerular pressure. However, these alterations begin to disappear after discontinuation of the treatment.

The histological results showed glomerular retraction, fibrosis, cellular debris, and an increase in urinary space between the Bowman’s capsule and the glomerulus. The tubules are also shrunken, having thickened tubular basement membranes, with variable interstitial fibrosis separating tubules, accompanied by perivascular fibrosis around the adventitia in the rats that consumed the 6% HSL infusion. Moreover, the effect of Ne on renal function is mediated through α_2_-adrenergic receptors by regulating the passage of intracellular Ca^2+^ after cAMP signaling. This stimulation causes contraction of the smooth muscle in the afferent arterioles, which had perivascular fibrosis, and this leads to an increase in the resistance in the renal blood vessels, resulting in an increase in the RVR, as observed when perfusing both Ne and Ach in the 6% HSL group. These results suggest that chronic consumption of 6% HSL modulates the increase in RVR in the kidney associated with anatomical changes, thus contributing to an increase in SBP and to a decrease in CCr. However, these alterations could begin to disappear after discontinuation of the treatment, and the rats were subsequently given tap water. In this regard, a recent study showed that chronic consumption of a 6% HSL infusion in rats led to RS and favored vasoconstriction, decreased relaxation, and increased thickness of elastic fiber layers associated with hypertrophy of the vascular wall in the thoracic aorta, which contributed to an increase in SBP [46].

Furthermore, HSL could provide excess tyrosine, the substrate for Ne synthesis by the sympathetic nerve endings, which could contribute to an increase in the SBP [47]. In spontaneously hypertensive rats, the consumption of an HSL infusion at 1000 mg/kg over 30–60 days also increased mortality in comparison with normotensive rats. This was associated with an increase in the uric acid level, serum albumin, and deposition of urate crystals in soft tissue and kidney stones [48]. In addition, ROS participation is well demonstrated in the renal system. They stimulate Na^+^ reabsorption, Cl^−^/HCO_3_^−^ exchange, Na^+^/K^+^ ATPase, the increase in Cl^−^ channels, tubule glomerular feedback in the macula densa, and in the collecting duct. They stimulate the activity of epithelial Na^+^ channels that cause dilation of the afferent arteriole and the K^+^ transport, which, in turn, increases tyrosine kinases and the synthesis of renal gluconeogenesis [49].

Another example of the beneficial participation of ROS is found in cardiac function. In mouse models with myocardial damage, NOX4 overexpression prevented H_2_O_2_ depletion and improved cardiac function after an ischemic period [50]. In this sense, our results show that the infusion of different ROS, such as KO_2_ and ONOO^−^, decreases the Δ-PP, but H_2_O_2_ favors an increase in the 6% HSL group when compared with groups C and HSL ± 6%. This indicates that the antioxidant enzyme overexpression is involved in the detoxification of these ROS. It is also associated with the excessive intake of antioxidants provided by the 6% HSL infusion that contributes to the reduction in these ROS, which are partly involved in modulating vascular reactivity. In this sense, H_2_O_2_ has vasodilatory effects on the arterioles. Furthermore, H_2_O_2_ activates 4-aminopyridine-sensitive K^+^ channels, which leads to the closure of Ca^2+^ voltage-dependent channels, producing hyperpolarization in arterial smooth muscle. Furthermore, H_2_O_2_ synthesis via the NOX4 pathway may contribute to RVR by increasing hyperpolarization of the vascular endothelial membrane in the kidney, which may promote activation of endothelial nitric oxide synthase (eNOS), thereby increasing nitric oxide (NO) synthesis [50]. Similarly, it is essential to maintain redox homeostasis in the endothelium since excessive production or decreased production of ROS contributes to impaired renal vascular function [13].

The ROS effects on vessels are not uniform, since they depend on which ROS is acting and at what concentration [51]. Therefore, our results suggest that O_2_^−^, ONOO^−^, and H_2_O_2_ participate in RVR modulation and that the excess of antioxidant molecules provided by the 6% HSL infusion decreases these ROS. After discontinuing the HSL treatment, this tended to normalize. In this regard, a recent study showed that when aortic rings of rats that consumed a 6% HSL infusion and were incubated in the presence of Ne and Ach, KO_2,_ vasoconstriction and vasodilation were normalized [46]. KO_2_ reacts violently with water to form KOH and, in this process, O_2_^−^ is formed. This ROS can act on renal function. Therefore, the present results highlight ROS participation in renal vascular response at normal concentrations. Furthermore, as previously mentioned, H_2_O_2_ promotes vasodilation and produces NO through the activation of different signaling pathways, such as PI3K/Akt, Erk1/2, and p38MAPK [52]. Recently, H_2_O_2_ was found not only to stimulate eNOS, but it can also increase its expression. Another protein involved in the regulation of vasodilation is PKG1α, a protein kinase that is sensitive to oxidation by H_2_O_2_ through the formation of a disulfide bond. The vasodilation that occurs is independent of cGMP levels [51]. The results obtained in our study show that the increase in Δ-PP in the 6% HSL group might involve the participation of H_2_O_2_ in the modulation of RVR. They also suggest that the excess of antioxidants provided by the HSL infusion decreases the H_2_O_2_ concentration, thereby altering vasorelaxation. However, these alterations may be reversible over time if the excess antioxidant intake is removed.

Furthermore, the O_2_^−^ anion can oxidize NO, generating ONOO^−^. At normal concentrations, these molecules can oxidize disulfide bridges (thiol groups) between protein cysteines. The balance between the oxidation and reduction processes in the thiol group content is very important since it regulates the activity and functionality of proteins and enzymes [17]. Our results showed that treatment with the 6% HSL infusion favored a decrease in the thiol group content compared to group C. However, when the treatment was replaced by plain water, the thiol group content increased. A predominant feature in RS is a decrease in thiol group content, a condition caused by misfolding of proteins in the endoplasmic reticulum, which leads to the accumulation of misfolded proteins with a loss of disulfide bridges in a state of stress. Furthermore, reticulum stress is caused by overstimulation of Nrf2 [53]. The results of our study show that the NrF2 phosphorylated expression is increased in the kidney homogenate in the group of rats that consumed the 6% HSL infusion compared to group C. In this regard, quercetin and resveratrol present in the HLS infusion may directly phosphorylate Nrf2 (post-translational modification) through several kinases, such as AMPK and PKC. This leads to increased stability, activity, and nuclear abundance of Nrf2, and this process increases the transcription rate of various antioxidant genes and detoxification enzymes. [54]. Another possibility may be the interaction of the anthocyanins present in the HLS infusion with the Keap1-Nrf2 pathway. This complex is then translocated to the nucleus, where it can directly regulate GSH levels by controlling the expression of the two subunits that make up the glutamate–cysteine ligase complex. Also, the anthocyanins may regulate the GR activity, which is maintained through the GSH/GSSG ratio for both the de novo synthesis and the recycling process [55]. Another study has demonstrated that the accumulation of misfolded proteins induces an alternative mechanism of Nfr2 activation through the autophagy of adapter p62, which competes with Keap-1 for the Nrf2 binding site, thus increasing its release, phosphorylation, and translocation to the nucleus [17]. p62 is also an Nrf2 target gene, creating a positive feed-forward activation loop [56]. This may lead to the overexpression of antioxidant phase II detoxification enzymes, such as GPx, SOD isoforms, CAT, GST, and TrxR, among others. This was also found in this work since the activities of these enzymes increased in the kidney homogenate of rats that consumed the 6% HSL infusion.

In addition, several studies in the cardiovascular system have demonstrated the role of the NO_3_^−^/NO_2_^−^ ratio in the inflammatory process and in RVR, suggesting that these agents can increase mitochondrial biogenesis when there is some injury to the vascular wall [13]. In this process, xanthine oxidoreductase can be overexpressed, which reduces these molecules to NO [14]. Furthermore, the excess antioxidants provided by the 6% HSL infusion can also reduce them to form NO, thus generating ONOO^−^ [57]. However, the overactivation of SOD isoforms may depress this molecule. In this sense, only a few studies have shown the beneficial effects that the ONOO^−^ could have on different cellular pathways. For example, low concentrations may induce the phosphorylation of band 3 tyrosine, a signaling pathway that activates glucose metabolism. Also, ONOO^−^ may cross the red blood cell membrane and react with hemoglobin, producing mainly metHb, which is reduced again to ferrous Hb by the reducing equivalents NADH^+^/NADPH^+^. Also, the low concentration of ONOO^−^ is catabolized by peroxiredoxins, leaving marginal levels of oxidative modifications in target biomolecules that can exert favorable redox signaling actions [57]. In this sense, the increased NO_3_^−^/NO_2_^−^ ratio concentration in the kidney homogenate of the rats that consumed the 6% HSL infusion indirectly suggests that there is overexpression/activity of iNOS or eNOS uncoupling. These situations are mainly present in proinflammatory states [58] and can be associated with the Δ-PP decrease when ONOO^−^ was perfused into the isolated kidney in the rats from the 6% HSL group. Under our conditions, ONOO^−^ is being detoxified by the overactivity of SOD isoforms [47]. Therefore, the beneficial effect of ONOO^−^ at low concentrations on RVR is demonstrated in this study. In this sense, ONOO^−^ transiently increases the reducing equivalents, promoting a protective response in the organism, improving the PPP pathway through the stimulation of the activity of G6PD, which increases NADPH^+^ production.

However, this mechanism can be harmful if maintained for a prolonged period of time [59]. G6PD is located in the vascular endothelium, and it is an enzyme with antioxidant effects responsible for providing NADPH^+^ and controlling ROS concentration [59]. In this regard, a study showed that G6PD overexpression decreased the excess ROS in endothelial cells treated with H_2_O_2_ or with tumor necrosis factor alpha. It also increased the GSH concentration [60]. In vascular cells, inflammatory processes, such as hypertrophy of the vascular wall, may increase G6PD expression associated with an increase in the activity of the PPP [61]. The results of our study show an increase in G6PD activity, which was significant in the group that consumed the 6% HSL infusion compared vs. group C. These results could be attributed to the increase in the RVR, which could lead to hypertrophy of the afferent arteriole wall, causing a proinflammatory process. However, further studies are required to confirm this hypothesis. A recent study showed that rats that consumed a 6% HSL infusion course in a proinflammatory state led to an RS state [61], and another study showed that there was an increase in G6PD activity in the thoracic aorta associated with thickening of the elastic fibers in the thoracic aorta [60]. Therefore, our results suggest that the excess antioxidants provided by the 6% HSL infusion may contribute to a proinflammatory process and to RVR alteration. This could activate the G6PD and generate an increase in the NADPH^+^ that is used by phase II detoxification enzymes [62], such as GPx and CAT, which are able to attenuate the harmful effects of H_2_O_2_ on blood vessels [63].

Our results show that the activities of CAT, GPx, and peroxidases were increased in the kidney homogenate in the group of rats that consumed the 6% HSL infusion. These enzymes detoxify and utilize H_2_O_2_ as a substrate, and in this process, they reduce it to H_2_O and molecular O_2_. In addition, HSL can provide Se [64] and favor an increase in the activities of the enzymes, depending on GPx and TrxR [65]. In this sense, a recent work reported that rats that consumed a 6% HSL infusion showed an increase in plasma Se, which was associated with an increase in the activities of GPx and TrxR, which neutralize H_2_O_2_. Moreover, TrxR reduces ascorbyl ROS by recycling ascorbate, in addition to participating in the regulation of gene expression of some transcription factors, such as NF-kβ, AP-1, p-53, the glucocorticoid receptor, and apoptosis regulatory kinase-1 [66]. TrxR is essential to maintain the homeostasis of thioredoxins, which are important thiol mediators in antioxidant systems, since they provide H^+^ protons through NADPH^+^ for the thiol group’s reduction. The results of the present study show a significant increase in the group that consumed the 6% HSL infusion in the activity of these enzymes. These results suggest that TrxR overactivity could be attributed to excess Se and that it constitutes a compensatory mechanism of the TrxR system attempting to increase the reduction in the thiol group content between protein cysteines, which were found to be decreased.

Likewise, various studies have observed synergistic effects between GSH and TrxR [67]. Therefore, high GSH concentrations could, in turn, favor an increase in TrxR activity [68]. Furthermore, as previously mentioned, TrxR depends on NADPH^+^, which is provided in part by the activity of G6PD, to generate more reducing equivalents. These equivalents are necessary for the altered functioning of this system associated with the increased percentage of the HSL infusion. On the other hand, GST is an antioxidant enzyme that conjugates GSH with electrophilic and xenobiotic agents, such as drugs, toxins, and carcinogens, and detoxifies the body [69]. The results of our study show a significant decrease in the activity of this enzyme in the kidney homogenate in the group of rats that consumed the 6% HSL infusion compared to groups C and HSL ± 6%. The administration of protocatechuic and tannic acids found in HSL at doses of 80 mg/kg reduces the activity of the mu, alpha, and pi subunits of GST in the liver [70]. Another study observed that the high concentrations of flavonoids also present in HSL could inhibit GST activity [71]. Therefore, the results suggest that lower GST activity could contribute to a decrease in the detoxification of some phenolic acids provided by HSL, thereby contributing to a positive feedback process that would continue perpetuating the concentration of these antioxidants systemically, favoring damage to renal physiology.

In another order of things, H_2_O_2_ is the product of SOD isoforms when detoxifying O_2_^−^ and NADPH oxidases; however, when there is an elevated activity of SODs, this produces an increase in H_2_O_2_ with a decrease in O_2_^−^, which can lead to an increase in the enzymes that detoxify it. This is in part due to the increase in the H_2_O_2_ substrate [71]. Our results show overactivity of the SOD-Mn and Cu-Zn isoforms in the kidney homogenate of the rats that consumed the 6% HSL infusion when compared to group C; however, when the treatment was replaced with plain water, this effect was normalized. These results suggest that the overactivity of the two SOD isoforms lowers the O_2_^−^ concentration since this ROS was significantly decreased. This is also evidenced by the irreversible oxidation of adrenaline to adenochrome in the kidney homogenate of the rats that consumed the 6% HSL infusion.

In addition to the abovementioned, H_2_O_2_ excess may be neutralized by the overactivity of the enzymes CAT, peroxidases, and GPx, which are predominantly localized in the renal tubules. This suggests that the overactivity of these enzymes generates harmful effects on renal physiology by decreasing or eliminating H_2_O_2_, which favors RS and leads to altered RVR, associated with physiological and anatomical changes in the kidney. In this regard, polyphenols, anthocyanins, cyanidin, and delphinidin-3-glucoside, which are provided by the HSL infusion, induce the activation of both SOD isoforms and GPx, acting as H^+^ proton donors in renal tissue. In addition, peroxidase activity was also increased in our experimental group treated with the HSL. Peroxidases, present in the renal tubules and in the glomerulus, can reduce H_2_O_2_ production by a reaction that resembles that of CAT [72], that is, by heterolysis. In this catalytic reaction, two protons are transferred from NADPH^+^ through the catalytic center of the enzyme, and H_2_O_2_ is used as a substrate to form important amounts of hypochlorous acid/hypochlorite in the presence of Cl^−^, in addition to H_2_O. This prevents the formation of the OH^−^ radical [73]. Therefore, the overexpression of this enzyme leads to a quantity of adducts in proteins and chlorinated lipids that cause dysfunction in the cells in different kidney compartments [74]. Therefore, these results suggest that the overactivity of CAT, GPx, and peroxidases contributes to the decrease in the H_2_O_2_ concentration, which participates in renal physiology when at normal concentrations.

**Figure 10:**
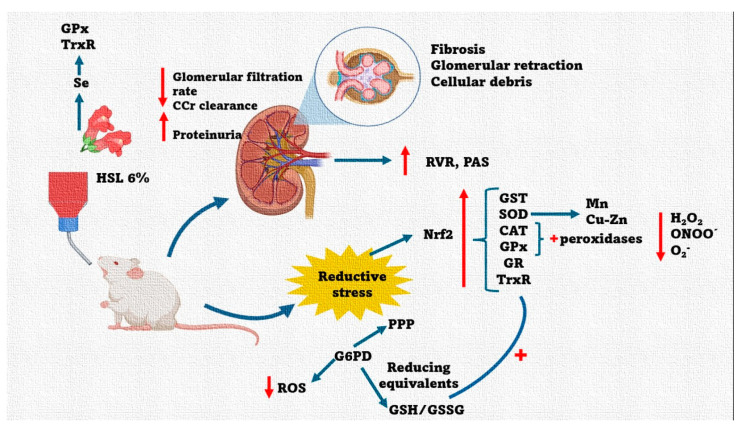
Summary of the physiological and anatomical changes in the kidney in response to a 6% HSL infusion. These changes are accompanied by alterations in the enzymatic and non-antioxidant systems that contribute to RS generation, which results in elevated systemic blood pressure in rats. Abbreviations: CAT = catalase, Nrf2 = nuclear factor erythroid 2-related factor 2, TrxR = thioredoxin reductase, GPx = glutathione reductase, GST = glutathione-S-transferase, SOD = superoxide dismutase, PPP = polyol pathway, CCr = creatinine clearance, G6PD = glucose-6-phosphate dehydrogenase, GR = glutathione reductase, GSH = glutathione, GSSG = oxidized glutathione, GST = glutathione-S-transferase, H_2_O_2_ = hydrogen peroxide, HSL = *Hibiscus Sabdariffa Linnaeus*, iNOS = inducible nitric oxide synthase, NADPH^+^ = nicotinamide adenine dinucleotide phosphate, NO_3_−/NO_2_^−^ = nitrate/nitrite ratio, O_2_^−^ = superoxide anion, PAS = systolic blood pressure, Se = selenium, up arrow = increase, and down arrow = decrease.

In addition, a characteristic redox couple affected in RS is the GSH/GSSG ratio. HSL extract can increase the GSH concentration by providing the amino acids cysteine, glycine, and glutamic acid. Our results show that the rats treated with 6% HSL showed a significant increase in the GSH/GSSG ratio when compared to group C. This increase may be due to the increased activity of G6PD, which provides the reducing equivalent NADPH^+^, which is then used as a cofactor by the GR selenoenzyme for the regeneration of GSH [60]. Our results show an increase in the activity of this enzyme. This suggests that the increase in GSH may be due to regeneration through the GR overactivity or by de novo synthesis using the amino acids provided by HSL through γ-glutamyl cysteine synthetase and glutathione synthetase. In this regard, increased GSH favors the mitochondrial membrane potential by increasing the reverse electron flow from succinate to NAD^+^ to increase ROS production by the mitochondria [74]. Moreover, the increase in the GSH/GSSG ratio could elevate the redox couple NADH/NADPH^+^ through G6PD and boost the electron transport chain in subunits I and III in O_2_^−^ production [72,73,74]. However, this increase would be paradoxical since it would lead to a state OS and not to RS. However, the O_2_^−^ anion can be depleted by the overactivity of the SOD isoforms. A summary of the results found in this paper is shown in Figure 10.

### Study Limitation

A limitation of this study is that the results provided are mainly descriptive, and it would be important to explore the mechanistic aspects of these effects in future publications. However, this study presents many novel results since reductive stress in the kidney has not been reported in a murine model due to chronic consumption of high concentrations of HSL.

## 5. Conclusions

Based on the results of this study, we conclude that excess antioxidants, such as flavonoids and polyphenols, among others, provided by a 6% HSL infusion increase the total antioxidant capacity. This capacity comprises the enzymatic and non-enzymatic antioxidant systems, resulting in an RS state that favors the increase in the redox couples GSH/GSSG and NADP^+^/NADPH^+^, thus contributing to decreasing ROS. This decrease impairs kidney function. However, the RS state can be reversed if the antioxidant intake provided by the HSL infusion is discontinued.

## Figures and Tables

**Table 1:** General characteristics and renal function markers in the groups of rats in the experimental groups.

Variables	HSL 6%	HSL ± 6%	C
Drinking water (mL/24 h)	13.4 ± 1.3 **	29.8 ± 2.7	25.4 ± 2.6
Weight of the right kidney (g)	1.2 ± 0.05	1.1 ± 0.05	1.2 ± 0.03
Urine (mL/24 h)	7.7 ± 0.8 *	21.1 ± 1.8	18.7 ± 4.5
CCr (mL/min)	0.05 ± 0.015 **	0.93 ± 0.29 *	1.61 ± 0.86
Albuminuria (mg/mL)	2.12 ± 0.30 **	0.98 ± 0.17	1.38 ± 0.26
Systolic blood pressure (mmHg)	145.2 ± 4.4 **	126.6 ± 2.1 *	118.7 ± 2.2
Body weight (g)	351.7 ± 17.0	341.0 ± 6.3	324.6 ± 6.9

** C and HSL ± 6% vs. HSL 6% *p* = 0.001, * C and HSL ± 6% vs. HSL 6% *p* = 0.01. Abbreviations: CCr = creatinine clearance.

**Table 2:** Markers in the non-enzymatic antioxidant system in the experimental groups.

Variables (mg the Protein)	HSL 6%	HSL ± 6%	C
GSH/GSSG ratio (μM)	0.024 ± 0.003 *	0.023 ± 0.002 †	0.016 ± 0.002
TAC (nM)	148.3 ± 21.9 **	136.6 ± 12.2 *	100.9 ± 8.4 †
NO_3_^−^/NO_2_^−^ ratio (nM)	0.19 ± 0.01 **	0.06 ± 0.02 **	0.04 ± 0.01 †
Thiols (μM)	74.6 ± 35.8 **	120.2 ± 10.5 **	142.4 ± 30.6 †

** C and HSL ± 6% vs. HSL 6% *p* = 0.01, * HSL ± 6% vs. HSL 6% *p* = 0.04 † C vs. HSL ± 6% *p* = 0.02.

## Data Availability

The datasets generated and analyzed during the current study are available from the corresponding author on reasonable request.

## Supplementary Materials

The following supporting information can be downloaded at: https://www.mdpi.com/article/10.3390/antiox14080984/s1, the perfused kidney and the enzymatic and non-enzymatic antioxidant system. References [75,76,77,78,79,80,81,82,83] are cited in the supplementary materials.

## Abbreviations

ROS	Reactive oxygen species
OS	Oxidative stress
GSH	Reduced glutathione
GSSG	Oxidized glutathione
O_2_^−^	Superoxide radical
O2	Molecular oxygen
H_2_O_2_	Hydrogen peroxide
ONOO^−^	Peroxynitrite
NO	Nitric oxide
OH^−^	Hydroxyl radical
CAT	Catalase
GPx	Glutathione peroxidase
RS	Reductive Stress
NADPH^+^	Reduced nicotinamide adenine dinucleotide
NADP^+^	Oxidized nicotinamide adenine dinucleotide
NAD	Nicotinamide adenine dinucleotide
G6PD	Glucose-6-dehydrogenase
Nrf2	Nuclear factor erythroid-derived 2
SOD	Superoxide dismutase
GST	Glutathione-s-transferase
GR	Glutathione reductase
Cu-Zn	Copper–zinc
Mn	Manganese
TrxR	Thioredoxin reductase
HSL	*Hibiscus sabdariffa Linnaeus*
Ne	Norepinephrine
Ach	Acetylcholine
SBP	Systolic blood pressure
SCr	Serum creatinine
CCr	Creatinine clearance
UCr	Urinary creatinine
KO_2_	Potassium superoxide
PP	Perfusion pressure
TAC	Total antioxidant capacity
RVR	Renal vascular resistance
PPP	Pentose phosphate pathway
NO_3_^−^/NO_2_^−^	Nitrates and nitrites
