# Supplementary material for: Impaired Renal Function Due to Reductive Stress Is Associated with Excessive Consumption of Hibiscus sabdariffa Linnaeus
Source: Antioxidants (Basel). 2025 Aug 11;14(8):984. doi: 10.3390/antiox14080984 (PMC12383063; doi:10.3390/antiox14080984)
Supplement: Supplementary file 1 [file antioxidants-14-00984-s001.zip › antioxidants-3751083-supplementary.pdf]

Supplementary material

1. Systolic Blood Pressure Measurement and Urine Collection

SBP was measured using a plethysmograph (Narco Bio-system) at the end of treatment and before euthanizing the animals. The rats were weighed and placed in metabolic cages after fasting for 24 hours before euthanasia. Urine was collected and stored at –30°C. The presence of albuminuria was determined using the bromocresol green method, and urinary creatinine (UCr) and serum creatinine (SCr) concentrations were determined to calculate creatinine clearance (CCr) [1].

2. Perfused kidney and the enzymatic and non-enzymatic antioxidant system

2.1 Isolated and Perfused Kidney

Rats were anesthetized by an intraperitoneal injection of sodium pentobarbital (63 mg/kg of body weight). The right kidney was exposed via a midline laparotomy, and the mesenteric and renal arteries and surrounding tissue were cleared. The right renal artery was cannulated via the mesenteric artery to avoid interruption of blood flow, and the kidney was removed, suspended, and perfused at a constant flow rate using a peristaltic pump (MasterFlex Easy-load II, no. 77200-50; Cole-Parmer Instrument Co., Vemon Hills, IL, USA) at 37°C and oxygenated with 95% O_2 and 5% CO_2 with Krebs solution at pH 7.4. The basal perfusion pressure (PP) was adjusted to 80–90 mmHg. The average flow rate of the perfusion solution was 8–9 mL/min. PP was measured with a transducer (Grass Telefactor, Grass Technologies, Astro Med, West Warwick, RI), coupled to a Grass model 79D polygraph and online software (Grass PolyView). Data are expressed as changes (Δ) in PP in millimeters of mercury (mmHg). After at least 15 min of perfusion and once a stable Δ-PP was obtained, vasoconstrictor responses to Ne and vasodilator responses to Ach were determined, both of which were given at intervals long enough to allow Δ-PP to return to baseline (between 75 and 90 mmHg). The bolus sequences and concentrations were: 20 μM Ne and 20 μM Ach, 2 μM potassium superoxide (KO_2), 1.7 μM hydrogen peroxide (H_2O_2), and 0.75 μM peroxynitrite ($ONOO^-$). Changes in PP due to Ne, Ach, and ROS were calculated by taking the mean of the pulsatile tracings before administration and the mean of the tracings at the peak Δ-PP value after administration. Data are expressed as changes in Δ-PP in mmHg. After each infusion bolus, the kidneys were left to recover for 20 min with Krebs solution to allow them to return to baseline Δ-PP (75–90 mmHg) and to detect signs of tachyphylaxis. The concentrations of Ne, Ach, and ROS were selected from published data, as they seemed the most suitable after obtaining changes in Δ-PP.

2.2*Histological Procedures to determne Anatomical Changes in the Kidney*

To demonstrate the anatomical and structural changes in the renal tissue, the left kidney was dissected and washed with 0.9% saline solution for 30 sec. The capsule was removed and cut in half. One half was processed for light microscopy according to standard techniques, which are briefly described below. One half of the kidney was fixed in 10% formalin solution for 24 hours, gradually dehydrated in ethanol, cleared in xylene, embedded in paraffin, and cut into 5 μM-thick slices using a microtome (Leica RM212RT, Wetzlar, Germany); the paraffin. Sections were stained with Masson's trichrome, Jones's methenamine and Sirius red techniques. Histological sections were analyzed at 25×, 12.5x and 32x respectively, according using a model 63300 optical microscope (Carl Zeiss, Oberkochen, Germany) equipped with a Tucsen digital camera (18 megapixels) coupled with TSview 7.3.1 software. The glomerular area was analyzed by densitometry using Sigma Scan Pro 5 Image

Analysis software (Systat Software Inc., San Jose, CA, USA). Density values are expressed in arbitrary pixel units. The other half of the kidney was homogenized in a cold sucrose buffer consisting of 25 mM sucrose, 10 mM Tris, 1 mM EDTA at pH 7.35 with protease inhibitors (1 mM PMSF, 2 μM pepstatin, 2 μM leupeptin, and 0.1% aprotinin). The homogenate was then centrifuged at 900× g for 10 min at 4°C, and the supernatant was separated and stored at -30°C until use. Total protein was determined using the Bradford method [2].

2.3 Determination of polyphenols, Total Flavonoids and Anthocyanins in HSL infusion

100 μL of the HSL infusion were diluted 1:10 in distilled water. Then, 500 μL of Folin-Ciocalteu reagent (2 N) were added, the mixture was homogenized, and incubated for 3 min at room temperature. Then, 3 ml of 2% sodium bicarbonate were added, the mixture was incubated for 15 min at room temperature, and the absorbance was read at 750 nm. The total flavonoids present in the HSL infusion were determined using the Jia method [3] and the absorbance was determined at 510 nm. The anthocyanins were determined according to the method described by Lee, and the absorbance was measured at 520 nm and 700 nm [4].

3. Determination of Non-enzymatic Antioxidant System Markers

3.1 NO_3^-/ NO_2^- ratio

To 100 μg of protein from the previously deproteinized kidney homogenate, 25 μL of 10% $ZnSO_4$ were added, then 25 μL of 0.5 N NaOH were added, the sample was homogenized and centrifuged at 5,000 rpm for 15 min. After centrifuging the samples, the supernatant was recovered and 50 μL of 0.14 M KH_2PO_4 at pH 7.35, plus 5 μL of NO_3^- reductase, were added, and the sample was incubated at 37°C for 45 min. Subsequently, 50 μL of 1% sulfanilamide were added, and the sample was incubated for 10 min in the dark. Then, 50 μL of 0.1% 1-naphthylethylenediamine were added, the total volume was adjusted to 1 mL. The absorbance was read at 540 nm [5-6].

3.2 GSH/GSSG ratio

To 100 μg of protein from the kidney homogenate, 100 μL of 0.05 M KH_2PO_4 pH 7.35, plus 100 μL of 5% Na_2WO_4) were added and then 100 μL of H_2SO_4 were added, the mixture was homogenized, and centrifuged for 5 min at 5000 rpm. The supernatant was recovered, and 700 μL of KH_2PO_4 plus 100 μL of 10 M Ellman's reagent were added. The mixture was incubated at room temperature for 5 min, and the absorbance was read at 412 nm [7]. To quantify GSSG, the procedure was the same as for quantifying GSH, but after deproteinizing the kidney homogenate, 4 μL of 8-vinylpyridine were added to oxidize the GSH present.

3.3 Determination of Total Thiol groups

100 μg of protein from the kidney homogenate were used, to which 100 μL of KBH4 dissolved in a 10 mM methanol/water (1:1 v/v) mixture were incorporated. The mixture was homogenized and incubated for 3 min. Then, 750 μL of buffer containing 6.7 mM formaldehyde, 10 mM EDTA, and 100 mM TRIS at pH 8.2 were incorporated and the mixture was incubated for 3 min. Then, 100 μL of 10 mM Ellman's reagent were added. The samples were incubated at room temperature for 4 min, and the absorbance was read at 415 nm [8].

3.4 Total Antioxidant Capacity

Evaluation of the total antioxidant capacity of the system (TAC) was performed by adding 1.5 mL of reaction mixture to 100 μg of protein from the kidney homogenate. The mixture consisted of 300 mM acetate buffer pH 3.6, 20 mM $FeCl_3$, 10 mM TPTZ in HCl in a ratio of 10:2:1 v/v respectively. The sample was mixed in the vortex for 5 sec and incubated at 37 ° C for 15 min in the dark, subsequently centrifuged at 5000 rpm. The absorbance was measured at 593 nm.

3.5 Superoxide Anion Detection

The O_2^- anion in kidney homogenates was determined by the irreversible oxidation of adrenaline to adenochrome as follows: 50 μg of protein were added to 2 mL of glycine buffer (50 mM) at pH 10.2, plus 50 μL of epinephrine (60 mM), and incubated and monitored at 30 °C for 6 min at 480 nm with an extinction coefficient of 4.0 mM^{-1} cm^{-1} [9].

4. Markers of the enzymatic antioxidant system

4.1 GST, GPx, GR and TrxR enzymes activities

To evaluate the activities of GR, GST, GPx, and TrxR, 100 μg of homogenized kidney, were used and treated as previously described [51]. All the samples were incubated and monitored at 340 nm, except the TxrR which was to 412 nm, by 6 min at 37°C. The GR activity was expressed as μmol of reduced GSSG/min/mg of protein, with an extinction coefficient of 6220 M^{-1} cm^{-1}. The GST activity was expressed as units of GS-TNB mol/min/mg of protein, with an extinction coefficient of 14,150 M^{-1} cm^{-1}. The GPx activity was expressed as nmol of $NADPH^+$ oxidized/min/mg of protein, with an extinction coefficient of 6220 $M^{-1}cm^{-1}$. The activity of TrxR is expressed as TNB nmol/min/mg of protein, with an extinction coefficient of 13,600 M^{-1} cm^{-1}.

4.2 SOD isoforms, CAT and peroxidases activities

The super oxide dismutase (SOD) isoform, catalase and peroxidase activities were determined through non-denaturing gel electrophoresis. 100μg of homogenized kidney were applied directly in non-denaturing 10% polyacrylamide gels. The electrophoresis was carried out at 120 volts for 4 h. For SOD isoform's activity, the gel was incubated with nitro blue tetrazolium at 2.45 mM for 20 min, then incubated with buffer of the KH_2PO_4 at 36 mM, EDTA at 28 mM, and riboflavin at 28 mM pH 7.8 (20 mL) and exposed for 10 min to UV light. Purified SOD from bovine erythrocytes with a specific activity of 112 U/mg of protein (Sigma-Aldrich, St. Louis, MO, USA) and purified was used as a control. For catalase activity the gel was incubated with a mixture of 1% $K_3Fe(CN)_6$ and 1% of $FeCl_3$ $6H_2O$ for 10 min in the dark and then washed with distilled water to stop the reaction. CAT from a bovine liver having a specific activity of 60 U/mg (Sigma-Aldrich) were used as positive control to calculate the activity of these enzymes. For the peroxidase activity, the gel was washed with distilled water three times, for 5 min, after being incubated with 3 mg/mL 3,3,5,5-tetramethylbenzidine dissolved in CH_3-OH/CH_3COOH/H_2O (1:1:1 *v/v*) with H_2O_2 (300 μL) total volume 20 mL, for 10 min. A quantity of 35 μL of horseradish peroxidase was loaded to a final concentration of 178.5 μg as a standard. The activities of the SOD isoforms, catalase and peroxidase gels were analyzed using densitometry with a Kodak Image® 3.5 system. The gels were analyzed by densitometry with image Sigma Scan Pro 5.1 software (Systat Software, Inc., San Jose, CA, USA). and are expressed as U of activity per mg of protein.

4.3 Glucose-6-phosphate dehydrogenase activity (G6PD)

G6PD activity was quantified using the kit provided by Sigma-Aldrich (MAR015-1KT) and, according to the manufacturer's specifications, 50 μg of protein from the kidney homogenate were used and monitored at a wavelength of 450–490 nm using a visible-light microplate reader (Stat Fax 3200 Awareness Technology, Palm City, FL, USA).

4.4 Nuclear factor erythroid 2 (NrF2)

50 μg of protein from the kidney homogenate were separated on an 8% SDS-PAGE gel and transferred to polyvinylidene difluoride membranes. The blot was blocked for 1 h at room temperature using Tris-buffered saline (TBS)-0.01% Tween plus 5% skim milk. The membranes were incubated overnight at 4 °C with the primary anti-phospho-NrF2-S40 monoclonal antibody produced in rabbit SAB5701902-100UL. The blot was then incubated with β-actin antibody (sc-81178) as a loading control. Images of the films were digitally acquired using a GS-800 densitometer with Quantity One software (Bio-Rad Laboratories, Inc., Hercules, CA, USA) and were reported as arbitrary units (AU).

References

1. BenGershom, E. Screening for albuminuria: A case for estimation of albumin in urine. *Clin Chem.* **1975**, *21*, 1795–1798.
2. Kruger, N.J. The Bradford method for protein quantitation Methods. *Mol. Biol.* **1994**, *32*, 9-15.
3. Jia, Z.; Tang, M.; Wu, J. The determination of flavonoid contents in mulberry and their scavenging effects on superoxide radicals. *Food. Chem.* **1999**, *64*, 555–559.
4. Lee, J.; Durst, R.; Wrolstad, R. Determination of total monomeric anthocyanin pigment content of fruit juices, beverages, natural colorants, and wines by the Ph Differential Method: collaborative study. *J. AOAC. Int.* **2005**, *88*, 1269–1278.
5. Tsikas, D. Analysis of nitrite and nitrate in biological fluids by assays based on the Griess reaction: Appraisal of the Griess reaction in the L-arginine/nitric oxide area of research. *J. Chromatogr. B. Analyt. Technol. Biomed. Life. Sci*. **2007**, *851*, 51–70.
6. Griess, J. Patents Relating to Chemistry:213,563. And 213,564 Coloring matters. *J. Am. Chem. Soc.***1879**, *1879*, 1.
7. Rahman, I.; Kode, A.; Biswas, S. Assay for quantitative determination of glutathione and glutathione disulfide levels using enzymatic recycling method. *Nat. Protoc.* **2006**, *6*, 3159–3165.
8. Erel, O.; Neselioglu, S. A novel and automated assay for thiol/disulphide homeostasis. *Clin. Biochem.* **2014**, *47*, 326–332.
9. Costa, V.M.; Silva, R.; Ferreira, L.M.; Branco, P.S.; Carvalho, F.; Bastos, M.L.; Carvalho, R.A.; Carvalho, M.; Remião, F. Oxidation process of adrenaline in freshly isolated rat cardiomyocytes: formation of adrenochrome, quinoproteins, and GSH adduct. *Chem. Res. Toxicol.* **2007**, *20*, 1183–1191.
